# Enhancing Radiotherapy Tolerance With Papaya Seed‐Derived Nanoemulsions

**DOI:** 10.1002/fsn3.70145

**Published:** 2025-04-04

**Authors:** Muhammad Tariq Siddiqui, Bilge Olceroglu, Zinar Pinar Gumus, Ahmet Murat Senisik, Firat Baris Barlas

**Affiliations:** ^1^ Vocational School of Health Services Altınbas University Istanbul Turkey; ^2^ Institute of Nanotechnology and Biotechnology Istanbul Univeristy‐Cerrahpasa Istanbul Turkey; ^3^ Department of Biotechnology Institute of Health Sciences, University of Health Sciences Turkey Istanbul Turkey; ^4^ Central Research Test and Analysis Laboratory Application and Research Center Ege University Izmir Turkey; ^5^ Clinical Research Excellence Application and Research Center Istanbul Univeristy‐Cerrahpasa Istanbul Turkey

**Keywords:** flovonoids, nanoemulsions, *Papaya carica*, radiation‐induced tissue damage, radioprotective agents

## Abstract

Flavonoid‐rich plant materials have gained attention for their potential to reduce radiotherapy side effects. *Carica papaya* (CP) seeds, known for high flavonoid content, hold promise for therapeutic applications. This study explored the extraction and evaluation of two oils—sunflower oil‐based papaya oil (SPO) and pure papaya oil (PPO)—and their nano emulsions (SPOE and PPOE), derived from CP seeds, for radioprotective effects. Chemical analysis using QTOF‐MS revealed antioxidants and phytochemicals in the oils and emulsions. Size analysis and zeta potential measurements using dynamic light scattering (DLS) showed particle sizes of 140 ± 26.06 nm for PPOE and 293.7 ± 49.42 nm for SPOE. Post‐radiation, both SPOE and PPOE significantly enhanced cell viability, with values of 72.24 ± 3.92 (*p* ≤ 0.001) and 75.85 ± 2.62 (*p* ≤ 0.001), respectively. These nanoemulsions show potential as topical agents for reducing radiation‐induced tissue damage in radiotherapy. Despite the promising in vitro findings, further in vivo studies are needed to confirm the clinical relevance of these nanoemulsions. Additionally, their incorporation into sunscreen formulations could provide further protection against radiation‐induced skin damage, broadening their potential applications.

## Graphical abstract

Flavonoid‐rich *Carica papaya* (CP) seeds were used to develop Sunflower Oil‐based Papaya Oil (SPO) and Pure Papaya Oil (PPO), alongside their nanoemulsions (SPOE and PPOE), for potential radioprotective applications. Post‐radiation testing on HaCaT cell lines demonstrated significant enhancement in cell viability, with PPOE and SPOE achieving 75.85% and 72.24%, respectively. These findings suggest their potential use as topical agents in radiotherapy and sunscreen formulations to mitigate radiation‐induced skin damage.

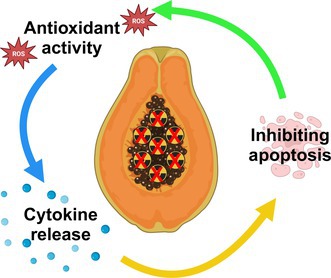

## 1 Introduction

Radiation therapy (radiotherapy) is a common and effective treatment method used for various types of cancer through the application of X‐rays (photons) (Hughes and Parsons 2020; Maddocks‐Jennings et al. 2005). Radiotherapy involves the application of ionizing radiation to malignant tissue to induce cell death and control tumor growth (Chen and Kuo 2017). It can be administered externally, internally, or systemically. External beam radiotherapy involves the use of radiation sources from outside the body. The required dose is delivered in fractions over a predetermined period, taking into account differences in repair and repopulation times between tumor cells and normal cells (Glover and Harmer 2014).

Approximately 50% of all cancer patients are treated with radiotherapy (Hughes and Parsons 2020). The major disadvantage of radiotherapy is the emergence of short‐ and long‐term adverse effects due to the irradiation of surrounding healthy tissues in order to deliver a lethal dose to cancerous cells (Moding et al. 2013; Siddiqui and Movsas 2017). Radiation exposure during therapy can cause damage to cellular membranes (Mishra 2004), modulate antioxidant enzymes (Hardmeier et al. 1997), and damage cellular DNA (Tiwari and Mishra 2020). Radiotherapy can lead to a range of side effects including fatigue, nausea, diarrhea, stiff joints and muscles, dry mouth, loss of appetite, and hair loss (Ingole et al. 2024). It is expected that approximately 87% of patients undergoing radiotherapy will experience moderate to severe skin reactions (Fisher et al. 2000). Protective treatments are implemented using various components such as radioprotectors to enhance the protection of healthy cells against radiation and radiosensitizers to sensitize cancer cells to radiation (Spalding and Lawrence 2006; Tiwari et al. 2010; Tiwari and Mishra 2020).

While numerous studies have been conducted to protect against the effects of radiation, the use of phytochemicals based on traditional methods has regained popularity (Aprotosoaie et al. 2016; Gumus et al. 2015). Various herbal agents such as curcumin and resveratrol have been extensively studied in studies for their potential radioprotective effects (Zhang et al. 2023). Agbele et al. (2020) study showed that resveratrol has antioxidant and anti‐inflammatory effects and prevents radiation‐induced damage. Qiao et al. (2012) study with the herbal agent curcumin reported its radioprotective effect. However, despite these promising findings, challenges remain in terms of bioavailability, stability, and clinical translation of these agents. In this context, *Carica papaya* seeds offer an alternative phytochemical source with a rich flavonoid profile, high antioxidant capacity, and potential for novel formulation approaches such as nanoemulsions to improve bioavailability and therapeutic efficacy. Compounds containing flavonoids obtained from nature, which reduce radiation damage or facilitate its healing, have become the focus of studies (Aprotosoaie et al. 2016). Flavonoids are plant metabolites that provide color pigments in various fruits and vegetables (Falcone Ferreyra et al. 2012). They are polyphenolic molecules containing 15 carbon atoms and are soluble in water (Tiwari and Mishra 2020). They offer substantial health benefits by modulating various cell signaling pathways and exhibiting antioxidant effects (Azarashkan et al. 2022; Pietta 2000). Flavonoids regulate numerous cellular signaling pathways that govern DNA damage and repair, cell cycle, and apoptosis, either alone or in combination with radiation. Flavonoids possess both radiosensitization and radioprotection properties and modulate several cellular pathways associated with the cell cycle and apoptosis (Falcone Ferreyra et al. 2012; Spalding and Lawrence 2006; Tiwari and Mishra 2020). Phytochemicals are natural compounds derived from plant sources and are thought to have the potential to alleviate the negative effects of radiotherapy in cancer treatment (Ranjan et al. 2019). These compounds generally have antioxidant properties and can help reduce cellular damage caused by free radicals (Abedinia et al. 2025; Soni and Sosa 2013; Zhang et al. 2015). Radiotherapy uses ionizing radiation to kill cancer cells. However, healthy tissue can also be damaged in the process. Phytochemicals protect healthy cells from radiation and increase the sensitivity of cancer cells to radiation, making treatment more effective (Jit et al. 2021).

*Carica papaya* (PC) is a herbaceous plant belonging to the Caricaceae family and is cultivated in tropical regions (Dotto and Abihudi 2021). PC is a tropical fruit‐bearing tree originating from southern Mexico, Colombia, and Venezuela (Latin America), but is now widespread in tropical and subtropical regions worldwide, including Pakistan, India, and Africa. Various components of the plant hold significant ethnomedical value, and traditional Indian practices are renowned for their medicinal benefits (Maruthanila et al. 2021). The chemical composition of PC fruit is complex, encompassing various chemical components found in different parts of the plant, such as enzymes, carbohydrates, fatty acids, minerals, vitamins, phenolics (including flavonoids and non‐flavonoids), terpenoids, alkaloids, and sulfur‐containing substances (Kaur et al. 2019; Zhou et al. 2011). Moreover, ripe papaya fruit is rich in iron, phosphorus, folate, calcium, and protein (Okon et al. 2017). Unripe fruit contains milky juice containing papain, an endolytic plant cysteine protease with various medical applications, serving as an antioxidant for the skin and beneficial in the treatment of inflammatory and chronic disorders (Kong et al. 2021). Papaya seeds and leaves have been associated with the management of diabetes and liver diseases, as well as with antitumor activities (Dotto and Abihudi 2021). The presence of concentrated phytochemicals such as flavonoids, phytosterols, carotenoids, alkaloids, phenolic compounds, and cyanogenic compounds (benzyl glucosinolate) in papaya seeds and leaves contributes to the plant's therapeutic effects (Doan et al. 2020; Odhong et al. 2014; Olcum et al. 2020; Singh et al. 2019). Recent studies have highlighted their potential to reduce oxidative stress and mitigate DNA damage caused by radiation exposure, making them promising candidates for radioprotection (Yogish Somayaji et al. 2016). Papaya seeds also increase membrane stability and cellular defense mechanisms against radiation‐induced lipid peroxidation (Kong et al. 2021). Given these biochemical properties, *C. papaya* seed oil represents a novel and understudied botanical candidate for radioprotective applications, distinguishing it from other widely studied phytochemicals.

In this study, *C. papaya* fruits were collected from Karachi, Pakistan. Subsequently, oil was extracted from the seeds of these fruits using two different methods, and nanoemulsions were synthesized from the extracted oils. Nanoencapsulation can enhance the oxidative stability of essential oils while also strengthening their antimicrobial effects (Gorzin et al. 2024). The content analyses, size analyses, and zeta potential measurements of these four formulations were conducted, followed by an evaluation of their cell‐based antioxidant analyses and radioprotective effects using a LINAC device. This study represents the first attempt in the literature to determine the radioprotective effects of cold press *C. papaya* seed oils, thereby unveiling their potential applications in various medical treatments and cosmetic products. This study seeks to deepen our understanding of biotechnologically modified herbal formulations and evaluate their potential to enhance the effectiveness of herbal ingredients. Compared to chemical antioxidants and radioprotective agents used in previous studies, nanoemulsions derived from papaya seeds offer a more natural and biodegradable alternative. The small particle size of nanoemulsions makes them a promising option for clinical, cosmetic, and industrial applications (Qi et al. 2021). Their potential to improve therapeutic efficacy and reduce radiation‐induced damage makes them a superior choice compared to traditional methods (Qi et al. 2021).

## 2 Material and Methods

### 2.1 Materials

In this study, all chemicals were used in analytical purity. Tween 60 (CAS: 9005‐67‐8), Phosphate Buffered Saline (PBS) (CAS: 524650‐1EA), Ethanol (EtOH), MTT (3‐[4,5‐dimethylthiazol‐2‐yl]‐2,5‐diphenyltetrazolium bromide) reagent (CAS: 57360‐69‐7), Sodium Dodecyl Sulphate (SDS) (CAS: 151‐21‐3), dimethyl sulfoxide (DMSO) (CAS: 67‐68‐5), 4′,6‐diamidino‐2‐phenylindole (DAPI) (CAS: 28718‐90‐3) and Fluorescein isothiocyanate (FITC) (CAS: 27072‐45‐3) were purchased from Merck (Germany). Distilled water was obtained using a Millipore purification system.

### 2.2 Collection of *C. papaya* Seeds

On January 31, 2023, ripe *C. papaya* (PC) fruits were collected from Karachi, Pakistan. Following the collection, all PC samples, including flowers, branches, leaves, seeds, and unripe fruits, were cataloged in the Herbarium of Altınbaş University under the code HERA 1230 on February 26, 2023 (Figure S1 and Figure S2). The PC fruits were subsequently cleaned with purified water, cut into pieces, and the seeds were extracted. These seeds were washed with purified water and sun‐dried to prepare for oil production.

### 2.3 Extraction of PC Seed Oil

PC seed oil was extracted using two different methods. In the first method, sunflower and PC seed oil (SOPO) were extracted from PC seeds preserved in sunflower oil, while in the second method, PC oil (PPO) was obtained solely from pure PC seeds. To extract the oil, 240 g of PC seeds were added to 1 L of sunflower oil purchased from the Karachi Empress Market, and the PC seeds were exposed to the sunflower oil for 6 days. Sunflower and PC seed oil (SPO) was obtained using the cold press technique. To extract SPO, the soaked seeds were placed into the extraction machine chamber. Secondly, pure PPO was obtained by cold‐pressing PC seeds directly in the extraction machine chamber. The cold press method was employed. The resulting PPO was then stored in an airtight bottle at room temperature and protected from light.

### 2.4 Emulsion Synthesis

PPOE and SPOE emulsions were formulated using PPO and SPO as the oil phases and Tween 60 (3%) as the surfactant. In these emulsion systems, Tween 60 was incorporated into the aqueous phase, while PPO and SPO constituted the oil phases. Oil‐in‐water (O/W) emulsions were prepared with a 60:40 (v/v) oil‐to‐water ratio, adjusting the specific proportions of the oil and water phases as necessary.

To obtain nanoemulsions, the appropriate amounts of oil and water phases were precisely weighed and subjected to ultrasonication using a probe at 60% amplitude for 5 min. To prevent potential heat‐induced degradation during the sonication process, the emulsions were maintained in an ice bath. The final emulsions were stored at +4°C until further analysis.

### 2.5 Chromatographic Analysis (QTOF‐MS)

A high‐resolution quadrupole time‐of‐flight mass spectrometer (QTOF‐MS) was employed for ionizing the chromatographic eluates, using an Agilent 6550 iFunnel Q‐TOF‐MS Accurate‐Mass system. This system featured an Agilent Dual Jet Stream electrospray ionization (Dual AJS ESI) interface, operating in both positive and negative ionization modes. The instrument parameters were optimized as follows: a drying gas flow rate of 14.0 L/min, a nebulizer pressure of 35 psi, a drying gas temperature set at 290°C, a sheath gas temperature of 400°C, and a sheath gas flow rate of 14 L/min, with nitrogen as the gas source. A nozzle voltage of 1000 V was applied. Spectral data were acquired in targeted MS/MS mode, covering a mass range from m/z 50 to 1800, with a collision energy of 20 eV for fragmentation during analysis (Sevimli‐Gur et al. 2021). Data processing and interpretation were conducted using the MassHunter Workstation software. The identification of phenolic compounds was facilitated by the MassHunter METLIN database and the Accurate Mass Personal Compound Database and Library (METLIN_AM_PCDL) provided by Agilent Technologies, Santa Clara, CA, USA.

### 2.6 Particle Size, Zeta Potential, and Short‐Term Size Depend Stability Analyze

For particle size and zeta potential measurements, DTS1070 disposable cuvettes were used for zeta potential analysis, while DTS0012 12 mm o.d. square disposable polystyrene cuvettes were utilized for particle size analysis. Prior to measurement, the samples were diluted at a 1:20 ratio in PBS. The particle size and zeta potential of the emulsions were then analyzed using dynamic light scattering (DLS) with a Zetasizer (Malvern, UK). Also, for short‐term size‐dependent stability analysis, the samples were stored at room temperature for 25 days, and size analyses were performed every 5 days using a Malvern DLS device (Figure S3).

### 2.7 Cell Culture

HaCAT cell line obtained from the American Type Culture Collection (ATCC). cells were grown in media containing Dulbecco's Modified Eagle Medium (DMEM) 10% fetal bovine serum (FBS) and 1.0% penicillin/streptomycin (Sigma Aldrich) in a 5.0% CO_2_ incubator at 37°C and 95% humidity under aseptic cell culture conditions. The cells were maintained by passaging twice per week.

#### 2.7.1 Cytotoxicity Assay

The MTT assay was performed following the method described by Barlas (2023). MTT (3‐(4,5‐dimethylthiazol‐2‐yl)‐2,5‐diphenyltetrazolium bromide) is a water‐soluble tetrazolium salt that is converted into water‐insoluble purple formazan by the succinate dehydrogenase enzyme in the mitochondria of living cells. For the assay, 8000 cells per well were seeded in 96‐well plates and incubated for 48 h under standard incubator conditions. Following incubation, the culture medium was removed and replaced with an application medium containing different formulations and concentrations of the test substances. All experimental groups, including controls, were prepared in the presence of 0.1% (v/v) DMSO, ensuring consistent solvent conditions across all treatments. At the end of the treatment period, the application medium was removed and replaced with 110 μL of MTT solution (10%, 5.0 mg/mL in PBS) per well, followed by incubation for 4 h. After incubation, 100 μL of SDS solution (1.0 g SDS in 10 mL PBS containing 0.01 M HCl) was added to dissolve the formazan crystals, and the plates were left in the incubator for 24 h. Absorbance was measured at 570 nm, with 630 nm as the reference wavelength, using a UV–Vis spectrophotometer. The results were analyzed by comparing absorbance values with the control group.

#### 2.7.2 Cell Based Oxidative Stress

In this study, a cell viability assay was utilized to evaluate the antioxidant effects of the treatments following H_2_O_2_‐induced oxidative stress (Guler et al. 2014). HaCaT cells were selected as the model cell line and cultured in 96‐well plates until reaching approximately 80% confluence. Seed oil and nanoemulsion samples were then applied to the wells, followed by a 2‐h incubation period. After pretreatment, the cells were washed with PBS to eliminate any residual nanoemulsions. To induce oxidative stress, the cells were subsequently treated with 1.25 mM H_2_O_2_ (diluted in DMEM) and incubated for 24 h under standard conditions (5% CO_2_, humidified atmosphere, 37°C). At the conclusion of the incubation period, cell viability was quantified using the MTT assay, and the results were analyzed in comparison to the untreated control group.

#### 2.7.3 Fluorescent Microscopy

Fluorescent microscopy relies on the unique property of fluorophores, which are molecules capable of absorbing light at a specific wavelength and emitting it at a longer wavelength. FITC was incorporated into the aqueous phase following the same procedure described previously. FITC was involved either in oil‐in‐water (O/W) or water‐in‐oil (W/O) emulsions within the aqueous phase. Excess FITC was removed by dialysis. The internalization of FITC‐labeled Emulsions into cells was assessed using a DM:4000B microscope equipped with a DFC7000T camera (LEICA, Germany). A total of 8 × 10^3^ cells were seeded onto a chamber slide and incubated for 48 h under standard cell culture conditions. After the incubation, the medium was replaced with treatment medium. The cells were further incubated for 2 h, followed by two washes with PBS. Fluorescent microscopy images were then captured under bright green and DAPI filter illumination (Tornaci et al. 2024), and the resulting images were merged using the ImageJ software.

#### 2.7.4 Radiotherapy

Cell irradiation for radiotherapy (RT) application was conducted as follows: 4 × 10^3^ cells were seeded into 96‐well plates and incubated overnight. Subsequently, the medium was removed, and the cells were treated with PPO, SOPO, POE, and SPOE (1.0 mg/mL) and incubated for 2 h. Following this, the cells were exposed to radiation doses of 0, 2, 4, and 8 Gray using a linear accelerator (LINAC) (Elekta Versa HD‐High‐Definition Dynamic Radiosurgery). This procedure has been previously described (Barlas et al. 2016). An effective radiation dose of 4 gray was also selected in the previous study by Barlas (2023). After irradiation, the cells were placed in an incubator at 37°C and 5.0% CO_2_ for 72 h. Cell viability was then assessed using a standard MTT assay, as previously outlined.

### 2.8 Statistical Analysis

The data in this study were reported as mean values accompanied by standard deviations (±SD). Statistical analyses were conducted using a one‐way analysis of variance (ANOVA), followed by Tukey's post hoc test for multiple comparisons. Each experiment was conducted at least three times to ensure reliability. Statistical significance was set at *p* values less than 0.05, while *p* values below 0.01 or 0.001 were considered highly significant.

## 3 Result and Discussion

### 3.1 Content Analysis

In recent years, there has been a growing interest in the study of natural compounds and their potential radioprotective effects (Maurya et al. 2006). This trend is primarily driven by the demand for effective agents capable of mitigating the adverse side effects of radiation exposure, particularly in patients undergoing radiotherapy for cancer treatment (Zhang et al. 2018). Natural compounds, such as polyphenols, flavonoids, and various plant‐derived extracts, have garnered significant attention due to their potent antioxidant and anti‐inflammatory properties, which can help shield normal cells from radiation‐induced oxidative damage (Jit et al. 2022). All content analyses of the emulsions were performed using Quadrupole Time‐of‐Flight Mass Spectrometry (QTOF‐MS), allowing for the precise identification and quantification of the active components (as shown in Tables 1 and 2). These analyses revealed that the SPOE and PPOE nanoemulsions are composed of a diverse array of bioactive compounds and phytochemicals, including flavonoids, phenolic acids, terpenoids, and other antioxidant‐rich substances. The differences observed among the tables in this study can be attributed to the use of sunflower oil as the infused oil. The results provide insight into how the use of an infused oil affects the content analysis, highlighting both the positive and negative impacts on the composition of the resulting product. This allows for a more accurate assessment of how the choice of infused oil influences the overall characteristics and efficacy of the final formulation.

**TABLE 1:** List of active ingredients found in SPOE as a result of Quadrupole time‐of‐flight mass spectrometry (QTOF‐MS) library scan.

Compound name	Rt	m/z	Polarity	Effect	Reference
L‐Arginine	1.081	175.1199	+, −	Antioxidant	Liang et al. (2018)
Uric acid	1.72	169.0366	−	Wound healing, antioxidant	Allen et al. (2004), Fernandez et al. (2014)
L‐Tyrosine	1.822	182.0826	−	Antioxidant, antidiabetic, anticancer, antimicrobial	İncili et al. (2025), Joondan et al. (2020)
Solanine	10.399	868.5117	+, −	Anticancer	Winkiel et al. (2022)
Carvone	14.048	151.1122	−	Antidiabetic, anti‐inflammatory, anticancer, antimicrobial, antiparasitic, immunomodulatory	Bouyahya et al. (2021)
Nobiletin	17.55	403.1393	−	Anti‐inflammatory, wound healing	Liao et al. (2018), Lien et al. (2016)
Piperine	18.595	286.1446	+, −	Anti‐aging, anti‐inflammatory, antimicrobial, anticancer, antiallergic, antidiabetic	Haq et al. (2021); Mujumdar et al. (1990)
Phytosphingosine	19.392	318.3015	+, −	Anti‐iflammatory, antimicrobial, Anti‐angiogenic	Kwon et al. (2007), Pavicic et al. (2007)
Dehydrocholic acid	22.861	425.2308	+, −	Antioxidant	Zhang et al. (2020)
Linoleic acid	23.052	281.2485	+, −	Antioxidant, wound healing	Fagali and Catalá (2008), Pereira et al. (2008)
Gulonic acid	1.215	195.0514	+, −	Wound healing	Yamashita et al. (2023)
Malic acid	1.316	133.0142	+, −	Antimicrobial	Raybaudi‐Massilia et al. (2009)
Citric acid	1.642	191.0204	+, −	Wound healing, antimicrobial	Chang et al. (2022); In et al. (2013)
L‐Phenylalanine	2.832	164.0718	+	Wound healing, anticancer	Xie et al. (2022); Yin et al. (2022)
Pyrocatechol	4.841	109.0295	+, −	Anti‐inflammatory	Funakoshi‐Tago et al. (2022)
Chlorogenic acid	5.93	353.0868	+	Antioxidant, anti‐inflammatory	Hwang et al. (2014); Sato et al. (2011)
Quinic acid	6.02	191.0564	+	Anticancer, wound healing	Genç et al. (2022); Murugesan et al. (2020)
Caffeic acid	6.525	179.0356	+	Antioxidant, anti‐diabetic, anti‐inflammatory, anticancer	da Cunha et al. (2004), Esmaeili et al. (2024), Kanimozhi and Prasad (2015), Xu et al. (2020)
Azelaic acid	10.343	187.0977	+, −	Anti‐inflammatory, antioxidant, antibacterial	Charnock et al. (2004), Sieber and Hegel (2014)
Isosteviol	18.707	317.2116	+, −	Antibacterial, Antifungal, Antibiofilm, Antioxidant, and Anticancer	Abdullah Al‐Dhabi et al. (2015), Hashmi (2023)

**TABLE 2:** List of active ingredients found in PPOE as a result of Quadrupole time‐of‐flight mass spectrometer (QTOF‐MS) library scan.

Compund name	Rt	m/z	Polarity	Effect	Referance
Gulonic acid	1.215	195.0514	+, −	Wound healing	Yamashita et al. (2023)
Malic acid	1.316	133.0142	+, −	Antimicrobial	Raybaudi‐Massilia et al. (2009)
Citric acid	1.642	191.0204	+, −	Wound healing, antimicrobial	Chang et al. (2022), In et al. (2013)
Pyrocatechol	4.841	109.0295	+, −	Anti‐inflammatory	Funakoshi‐Tago et al. (2022)
Azelaic acid	10.343	187.0977	+, −	Anti‐inflammatory, antioxidant, antibacterial	Charnock et al. (2004), Sieber and Hegel (2014)
Traumatic acid	14.68	227.1287	−	Anti‐inflammatory, anticancer, wound healing	Jabłońska‐Trypuć et al. (2019), Siracusa et al. (2018), Teng et al. (2018)
Isosteviol	18.707	317.2116	+, −	Antibacterial, Antifungal, Antibiofilm, Antioxidant, Anticancer	Abdullah Al‐Dhabi et al. (2015), Hashmi (2023)
Capric acid	20.698	171.1393	−	Antibacterial, anti‐inflammatory	Huang et al. (2014)
Stearic acid	31.566	283.264	−	Wound healing, anti‐inflammatory	Pan et al. (2010), Su et al. (2019)
L‐Arginine	1.081	175.1199	+, −	Antioxidant	Liang et al. (2018)
Solanine	10.399	868.5117	+, −	Anticancer	Winkiel et al. (2022)
Stearidonic acid	14.779	277.2174	+	Anti‐inflammatory	Sung et al. (2017)
Piperine	18.595	286.1446	+, −	Anti‐aging, anti‐inflammatory, antimicrobial, anticancer, antiallergic, antidiabetic	Haq et al. (2021), Mujumdar et al. (1990)
Phytosphingosine	19.392	318.3015	+, −	Anti‐iflammatory, antimicrobial, Anti‐angiogenic	Kwon et al. (2007), Pavicic et al. (2007)
Dehydrocholic acid	22.861	425.2308	+, −	Antioxidant	Zhang et al. (2020)
Linoleic acid	23.052	281.2485	+, −	Antioxidant, wound healing	Fagali and Catalá (2008), Pereira et al. (2008)

### 3.2 Particle Size

The study provided valuable insights into the intricate relationship between nanoemulsions and cellular interactions, emphasizing parameters such as zeta potential and other physicochemical characteristics. Research has highlighted that particle size is a crucial determinant of nanoemulsion stability, as it directly influences properties such as encapsulation efficiency and drug release profile (Danaei et al. 2018). These properties, in turn, significantly impact the efficiency with which particles are internalized by cells. For example, a study by Kulkarni et al. demonstrated that particles with a size of approximately 100 nm are more efficiently internalized by cells (Kulkarni and Feng 2013). Surface charge also plays a pivotal role in this process. Studies have shown that liposomes with a surface charge exhibit markedly enhanced cellular uptake, with evidence suggesting that charged liposomes can achieve nearly 20‐fold higher internalization compared to their neutral counterparts (Düzgüneş and Nir 1999). In this study, nanoemulsions derived from the *C. papaya* plant were characterized using DLS and zeta potential measurements, performed with a Malvern Zetasizer Nano ZS model. The particle sizes of PPOE and SPOE were determined to be 140 ± 26.06 nm and 293.7 ± 49.42 nm, respectively. Additionally, the zeta potential measurements revealed values of 7.1 ± 3 mV for PPOE and 12.6 ± 2 mV for SPOE (Table 3). These parameters play a critical role in understanding the stability, cellular interaction, and uptake potential of the nanoemulsions, contributing to their overall efficacy in biological applications.

**TABLE 3:** Size distribution and zeta potential of plane PPOE and SPOE.

	Size (nm)	Polydispersity index	Zeta potential (mV)
PPOE	140 ± 26.06	1.00	7.1 ± 3
SPOE	293.7 ± 49.42	1.00	12.6 ± 2

### 3.3 Cell Viability

**FIGURE 1:**
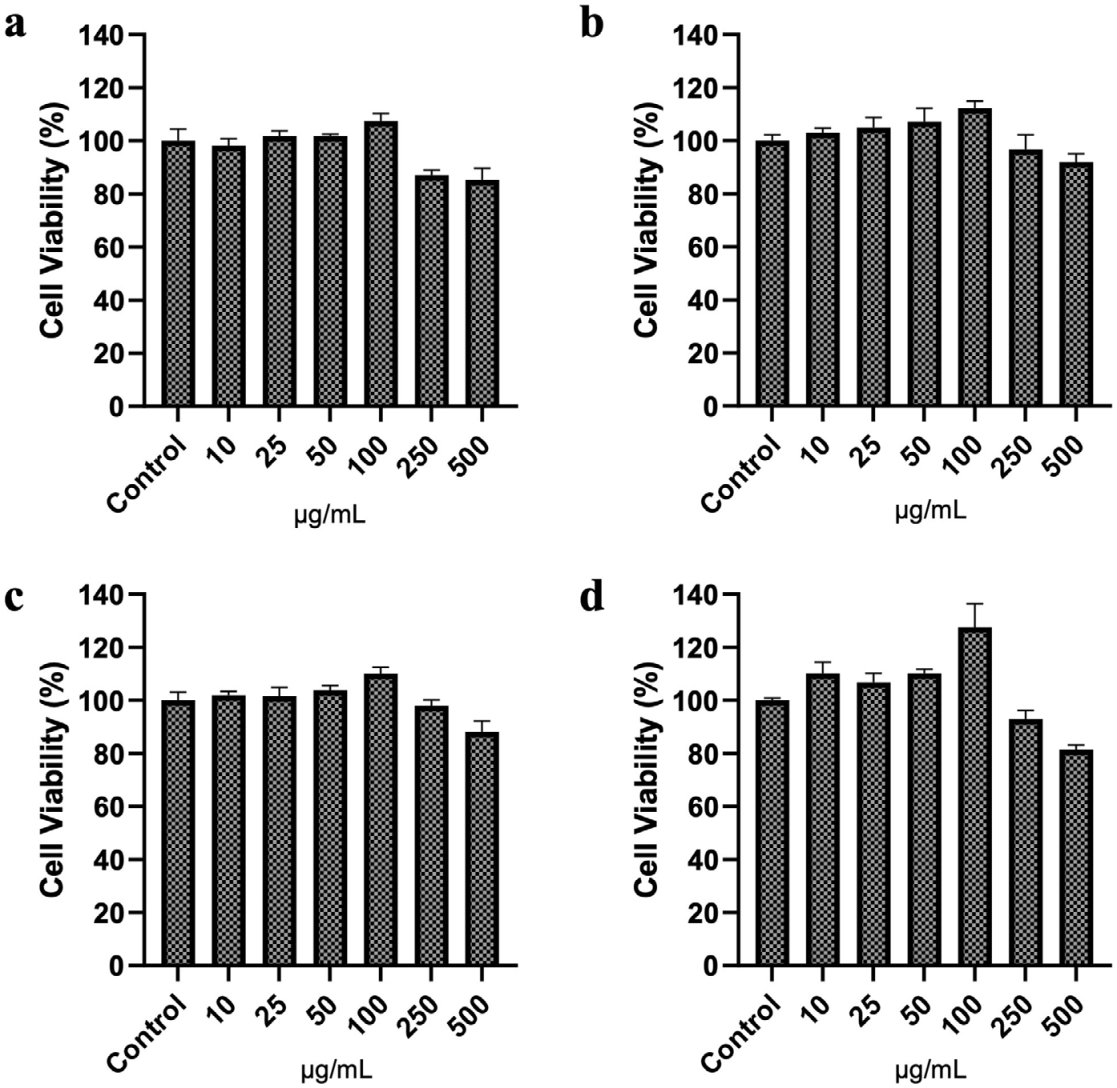
In vitro cytotoxic effect of seed oil and their emulsions: (a) SPO, (b) PPO, (c) SPOE, and (d) across the HaCaT cell line at 72 h. No significant difference was observed between the groups (ns).

The objective of this study is to highlight the radioprotective potential of emulsions and oils derived from *C. papaya* seeds. To this end, four different samples—SPO, PPO, SPOE, and PPOE—obtained from *C. papaya* plants were evaluated for their cytotoxic effects at six different concentrations (10, 25, 50, 100, 250, and 500 μg/mL) on the HaCaT cell line, with incubation for 72 h under standard cell culture conditions. Following the incubation period, cell viability was assessed using the MTT assay. Figure 1 presents the viability studies for SPO, PPO, SPOE, and PPOE. The results demonstrated that the samples obtained from *C. papaya* seeds, including SPO, PPO, SPOE, and PPOE, contributed positively to cell proliferation. Notably, in the studies conducted on the HaCaT cell line, the nanoemulsions derived from *C. papaya* at a concentration of 100 μg/mL exhibited the highest cell viability across all groups. At 100 μg/mL concentrations, MTT results for SPO, PPO, SPOE, and PPOE were measured as 107.37 ± 2.91; 112.34 ± 2.68; 110.02 ± 2.54; 127.59 ± 8.88, respectively. The radioprotective activity of phytochemicals is (100 μg/mL) was selected as the working dose for further experiments. Although the present study demonstrates the short‐term cytocompatibility of SPOE and PPOE nanoemulsions in HaCaT cells, the long‐term cytotoxicity and potential bioaccumulation effects remain to be investigated. Future studies should include prolonged exposure assays, apoptosis/necrosis analysis, and in vivo biocompatibility assessments.

### 3.4 Cell‐Based Oxidative Stress

**FIGURE 2:**
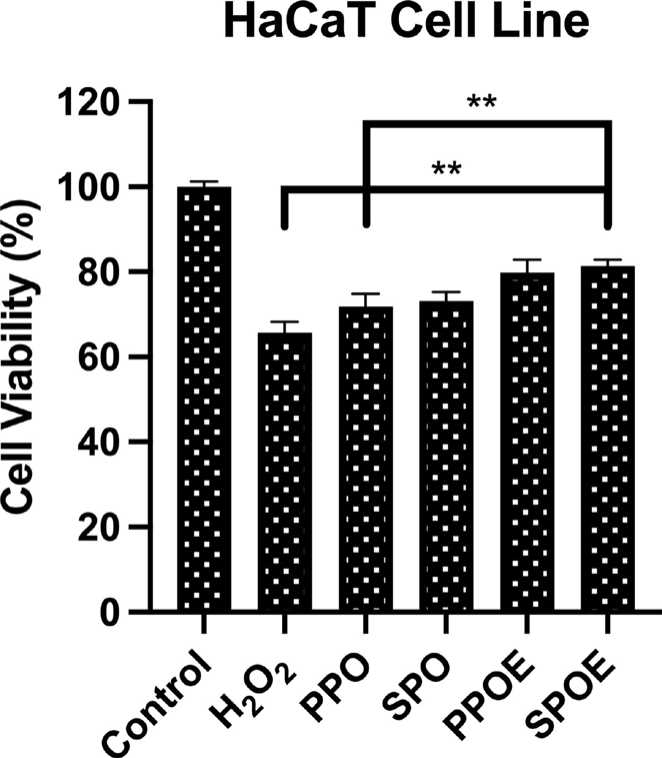
Effect of seeds oil and their emulsions form on hydrogen peroxide‐induced oxidative stress on HaCaT cell line. The mean is accompanied by the standard deviation (±SD). Statistically significant variations are indicated with asterisks (**p < 0.01).

The antioxidant activity of phytochemicals is mediated through various mechanisms, including the scavenging of free radicals, enhancement of antioxidant status, and the elevation of anti‐lipid peroxidation potential (Salehi et al. 2021). In this study, the hydrogen peroxide method was utilized to assess the antioxidant capacity of core oils and their emulsion derivatives under in vitro conditions. Cells were pre‐treated with the samples for 2 h, followed by exposure to hydrogen peroxide to induce oxidative stress. Cell viability was measured 24 h post‐treatment using the MTT assay. The results indicated that both SPOE (81.34 ± 1.51) and PPOE (79.83 ± 2.99) significantly maintained cell viability compared to the control group (Figure 2). This effect can be attributed to the presence of various phenolic hydroxyl groups bound to aromatic ring structures. Polyphenols, particularly flavonoid glycosides, isoflavones, and their derivatives (as shown in Tables 1 and 2), contain ketone groups conjugated to aromatic rings. These structures exhibit activity through electron‐donating groups, which inhibit energy transfer within cells, thereby reducing oxidative stress and stabilizing redox processes. Beyond flavonoids, content analysis revealed the presence of compounds such as azelaic acid, alpha‐linoleic acid, and L‐arginine, which possess both antioxidant and anti‐inflammatory properties, further contributing to the observed effects (Ponnampalam et al. 2019; Spaggiari et al. 2023).

### 3.5 Fluorescent Imaging

**FIGURE 3:**
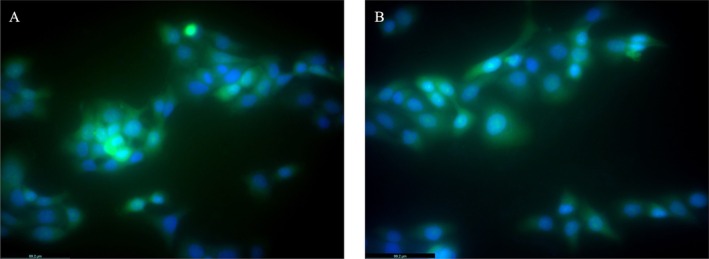
Fluorescence microscope images of the FITC and DAPI labeled nanoemulsions with HaCaT cell line. (A) PPOE (B) SPOE. Scale bar is 69.2 μm.

Fluorescent labeling has emerged as a robust alternative to radioactive labeling, providing a reliable means for monitoring the localization of nanocarrier systems. Fluorescent imaging, with its capacity for high‐resolution analysis at the cellular level, has become a critical tool in biomedical research (Faghihi et al. 2023). In this study, nanoemulsions were conjugated with FITC to facilitate the evaluation of their cellular uptake and subsequent intracellular localization. The experimental findings revealed that both nanoemulsion derivatives were successfully internalized by HaCaT cells, exhibiting a predominant localization within the cytoplasm (Figure 3). These outcomes suggest that the nanoemulsions are capable of delivering encapsulated phytochemicals effectively into the cytoplasm, thereby offering potential support to cellular defense mechanisms. Moreover, the results hold significant implications for advancing applications in radiotherapy and studies involving cellular oxidative stress.

### 3.6 Radiotherapy

**FIGURE 4:**
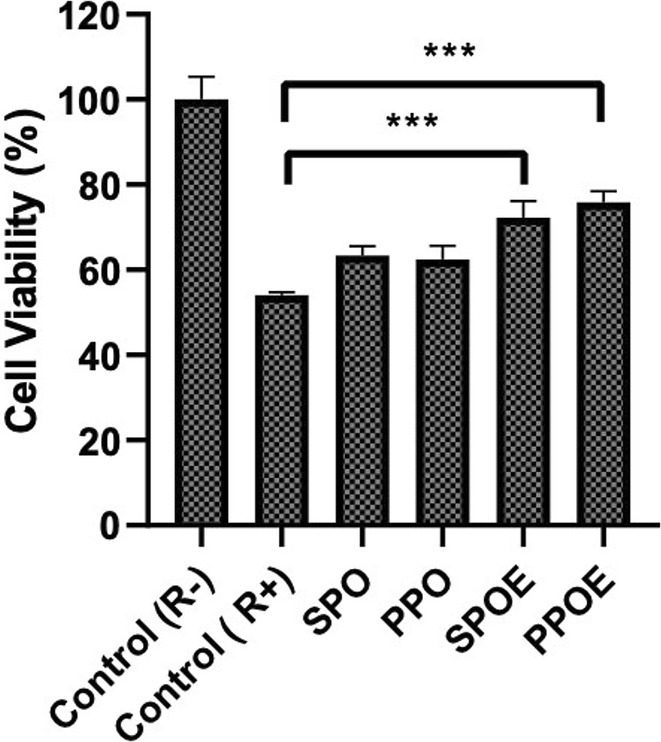
MTT results for Radioprotective effect of seed oils and emulsions under 4 Gray radiation. (R−) mean is no radiation and (R+) mean is 4 Gy Radiation. The mean is accompanied by the standard deviation (±SD). Statistically significant variations are indicated with asterisks (***p < 0.001).

The effectiveness of radiotherapy in treatment is accepted worldwide, and there are many applications for this treatment method especially according to DIRAC (Directory of Radiotherapy Centres) and GLOBOCAN, combined with the assumption that over 50% of all cancer patients would require Radiotherapy (Abdel‐Wahab et al. 2024; Borras et al. 2016). On the other hand, studies have shown that radiotherapy causes damage to healthy cells (P. Singh et al. 2024). In this study, a suitable concentration of 100 μg/mL of SOPO, PPO, SPOE, and PPOE was administered to the HaCaT cell line in 96‐well plates 2 h prior to irradiation (pre‐treatment). Following pre‐treatment, the cells were exposed to irradiation at a dose of 4 Gray using the Elekta Versa HD linear accelerator (Elekta AB, Stockholm, Sweden). The irradiation process utilized 6 MV photon beams, with a 0° gantry angle, a field size of 40 cm × 40 cm, and a focus‐to‐skin distance of 98 cm. After irradiation, the cells were incubated for 72 h, and cell viability was subsequently assessed using the standard MTT assay. The results indicated that both SPOE (72.24 ± 3.92, *p* ≤ 0.001) and PPOE (75.85 ± 2.62, *p* ≤ 0.001) demonstrated significant radioprotective effects on the HaCaT cell line, particularly at the 4 Gray radiation dose (Figure 4). These findings suggest that the pre‐treatment with SPOE and PPOE could help mitigate the adverse effects of radiation on cellular viability. It is important to note that the impact of radiation on cells can extend beyond immediate effects, as late normal tissue side effects may manifest several months or even years after radiation exposure. These delayed effects underscore the need for continued investigation into radioprotective agents to improve the long‐term outcomes of radiotherapy treatments (Bentzen 2006; De Ruysscher et al. 2019). These side effects are typically chronic and progressive, often resulting in a significant reduction in the quality of life for patients following treatment (Bentzen 2006). As a result, these side effects are often considered when determining safe radiation exposure limits (Bentzen 2006). In contrast to early side effects, the onset of late tissue reactions is influenced by a range of physiological factors, including radiation dose, cellular senescence, chronic inflammation, hypoxia, and fibrosis. Each of these factors contributes to the impairment of the tissue's regenerative capacity (Bentzen 2006). Fibrosis, in particular, contributes to the development of adverse effects in various tissues, including the heart, lungs, and liver (Bentzen 2006; Groarke et al. 2014; McDonald et al. 1995). The radioprotective activity of phytochemicals is mediated through various mechanisms, including the scavenging of free radicals, enhancement of antioxidant status, and increased anti‐lipid peroxidation potential. This activity is primarily attributed to the presence of phenolic hydroxyl groups attached to aromatic ring structures. Polyphenols, particularly flavonoid glycosides, isoflavones, and their derivatives contain ketone groups conjugated to aromatic rings. These structures become active through electron‐donating groups, which inhibit energy transfer within cells, thereby reducing oxidative stress and stabilizing redox processes. The literature has documented that flavonoids possess significant antioxidant activity, contributing to the prevention of various cancers by inducing apoptosis and subsequent cell death, thereby offering potential benefits in radioprotective applications (Abotaleb et al. 2018). Many phytochemicals were identified in the studied SPOE and PPOE, as detailed in Tables 1 and 2. These phytochemicals are well‐documented in the literature for their potent antioxidant effects, which contribute to their potential radioprotective properties (Hazra et al. 2012; Zhang et al. 2023). They demonstrate radioprotective effects not only by providing antioxidant support to cells but also by modulating key biochemical pathways. This includes upregulating DNA repair genes, inducing cytokine release, enhancing cellular antioxidant activity, and inhibiting apoptosis, thereby offering a multifaceted approach to radioprotection (Dowlath et al. 2021). The findings of this study indicate that the antioxidant and radioprotective properties of these compounds hold promise for potential applications in various topical formulations, such as sunscreens or skin protectants, designed to mitigate oxidative stress and protect the skin from radiation‐induced damage. The encouraging in vitro results demonstrating radioprotective effects necessitate further investigation into the long‐term efficacy of these compounds in shielding the skin from radiation exposure, as well as their practical applications. However, prior to clinical or commercial evaluation, comprehensive research is required to assess their safety, bioavailability, and overall effectiveness in such formulations.

## 4 Conclusion

This study highlights the promising potential of *C. papaya* seed‐derived oils and emulsions, specifically Sunflower Oil‐based Papaya Oil (SPO) and Pure Papaya Oil (PPO), along with their respective emulsions (SPOE and PPOE), as effective radioprotective agents. The comprehensive analysis of their chemical compositions through QTOF‐MS and the characterization of their physicochemical properties via DLS revealed a diverse range of antioxidant‐rich phytochemicals. The results from the biological evaluations, including cell viability assays and fluorescent imaging, demonstrated that both SPOE (72.24 ± 3.92, *p* ≤ 0.001) and PPOE (75.85 ± 2.62, *p* ≤ 0.001) significantly enhanced cell survival in HaCaT cells exposed to ionizing radiation. In conclusion, these findings suggest that the nanoemulsions not only protect against radiation‐induced oxidative stress but also offer potential as topical agents to reduce the adverse effects of radiotherapy. No significant difference was observed between the use of pure papaya seed oil (PPOE) and its infused form (SPOE) in the conducted experiments. Additionally, their formulation into products such as sunscreens could provide enhanced protection against environmental radiation exposure. However, while these results are promising, several limitations should be noted. In vivo studies are necessary to evaluate the long‐term effects and biosafety of emulsions derived from the *C. papaya* plant. Additionally, it will be important to assess how these nanoemulsions perform in real‐world clinical settings, considering factors such as bioavailability, stability, and individual responses. Overall, the study contributes to the growing body of research on natural radioprotective compounds, paving the way for future investigations into their broader applications in clinical and cosmetic formulations.

## Author Contributions

**Muhammad Tariq Siddiqui:** conceptualization (equal), data curation (equal), formal analysis (equal), investigation (equal), methodology (equal), project administration (equal), software (equal), validation (equal), visualization (equal), writing – original draft (equal), writing – review and editing (equal). **Bilge Olceroglu:** data curation (equal), methodology (equal), software (equal), supervision (equal), validation (equal), visualization (equal), writing – original draft (equal), writing – review and editing (equal). **Zinar Pinar Gumus:** data curation (equal), investigation (equal), resources (equal), software (equal), supervision (equal), validation (equal), visualization (equal), writing – original draft (equal), writing – review and editing (equal). **Ahmet Murat Senisik:** conceptualization (equal), data curation (equal), formal analysis (equal), funding acquisition (equal), investigation (equal), methodology (equal), project administration (equal), resources (equal), software (equal), supervision (equal), validation (equal), visualization (equal), writing – original draft (equal), writing – review and editing (equal). **Firat Baris Barlas:** conceptualization (equal), data curation (equal), formal analysis (equal), funding acquisition (equal), investigation (equal), methodology (equal), project administration (equal), resources (equal), software (equal), supervision (equal), validation (equal), visualization (equal), writing – original draft (equal), writing – review and editing (equal).

## Conflicts of Interest

The authors declare no conflicts of interest.

## Supporting information


**Figure S1.** The images of the papaya fruit, seeds, and flowers.
**Figure S2.** Papaya plant and herbarium records.
**Figure S3.** Short‐term size depends on stability analyze. The samples were stored at room temperature.

## Data Availability

The data that support the findings of this study are available from the corresponding author upon reasonable request.
