# Supplementary material for: Enhancing Radiotherapy Tolerance With Papaya Seed‐Derived Nanoemulsions
Source: Food Sci Nutr. 2025 Apr 4;13(4):e70145. doi: 10.1002/fsn3.70145 (PMC11971051; doi:10.1002/fsn3.70145)
Supplement: Supplementary file 1 — Figure S1. The images of the papaya fruit, seeds, and flowers. Figure S2. Papaya plant and herbarium records. Figure S3. Short‐term size depends on stability analyze. The samples were stored at room temperature. [file FSN3-13-e70145-s001.docx]

**Supplementary Info**

**Enhancing Radiotherapy Tolerance with Papaya Seed-Derived Nanoemulsions**

*Muhammad Tariq SIDDIQUI^1^, Bilge OLCEROGLU^2,3^, Zinar Pinar GUMUS^4^, Ahmet Murat SENISIK^1*^, Firat Baris BARLAS^2,5*^*

^1- Vocational School of Health Services, Altınbas University, 34217, Istanbul, Turkey.^

^2- Institute of Nanotechnology and Biotechnology, Istanbul Univeristy-Cerrahpasa, 34500, Istanbul, Turkey.^

^3-^ ^Department of Biotechnology, Institute of Health Sciences, University of Health Sciences Turkey, 34668 Istanbul, Turkey.^

^4-^ ^Central Research Test and Analysis Laboratory Application and Research Center, Ege University, 35100 Izmir, Turkey.^

^5- Clinical Research Excellence Application and Research Center, Istanbul Univeristy-Cerrahpasa, 34098, Istanbul, Turkey.^

Figure S1:


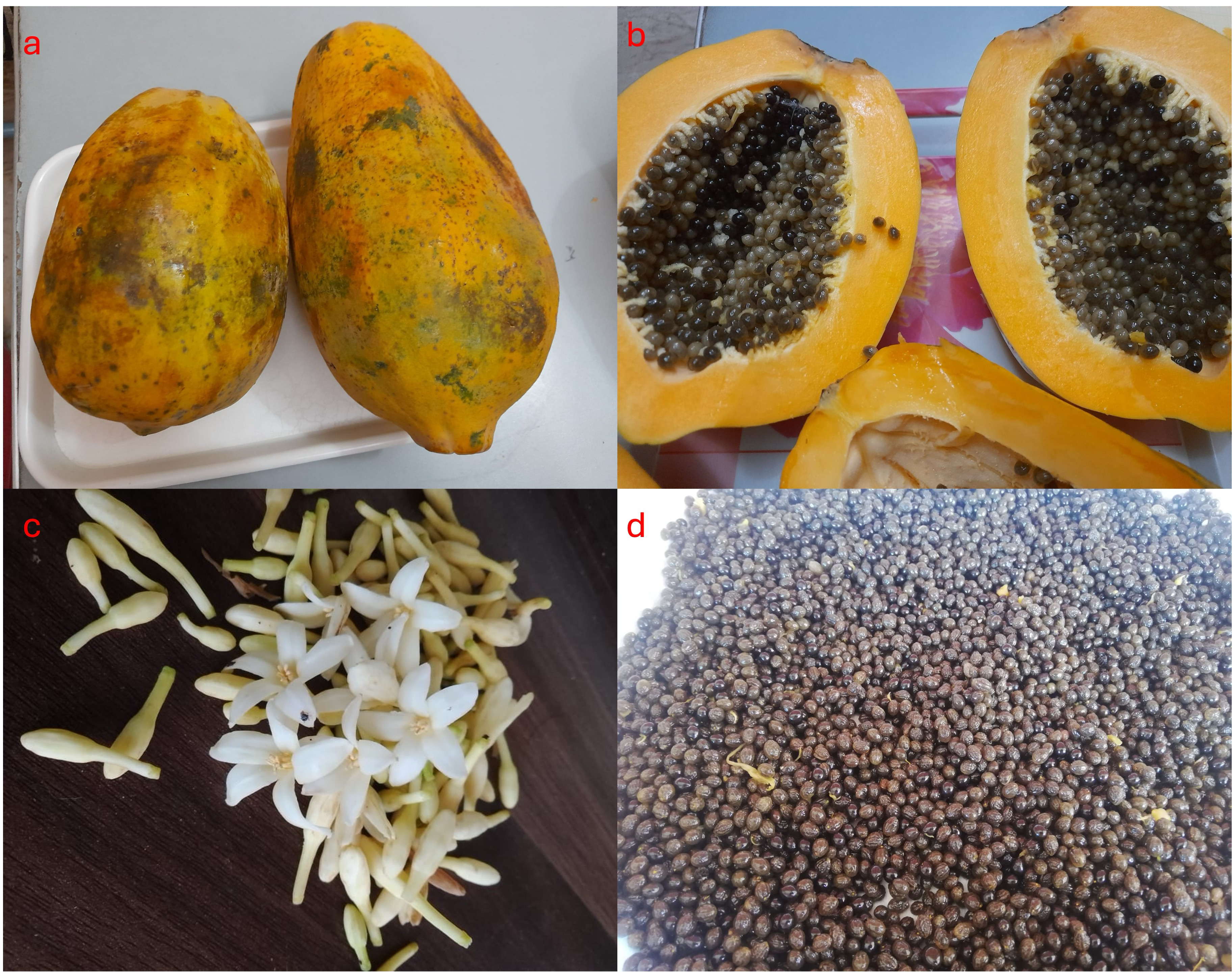


Figure S1. The images of the papaya fruit, seeds, and flowers.

Figure S2.


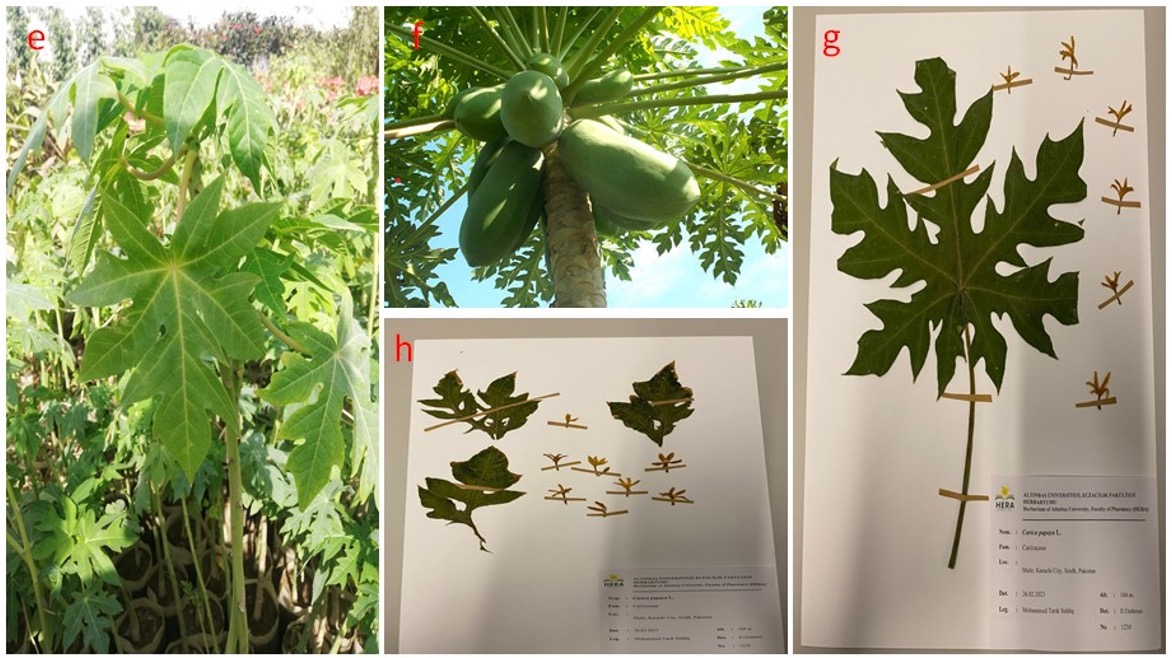


Figure S2. Papaya Plant and Herbarium records

Figure S3


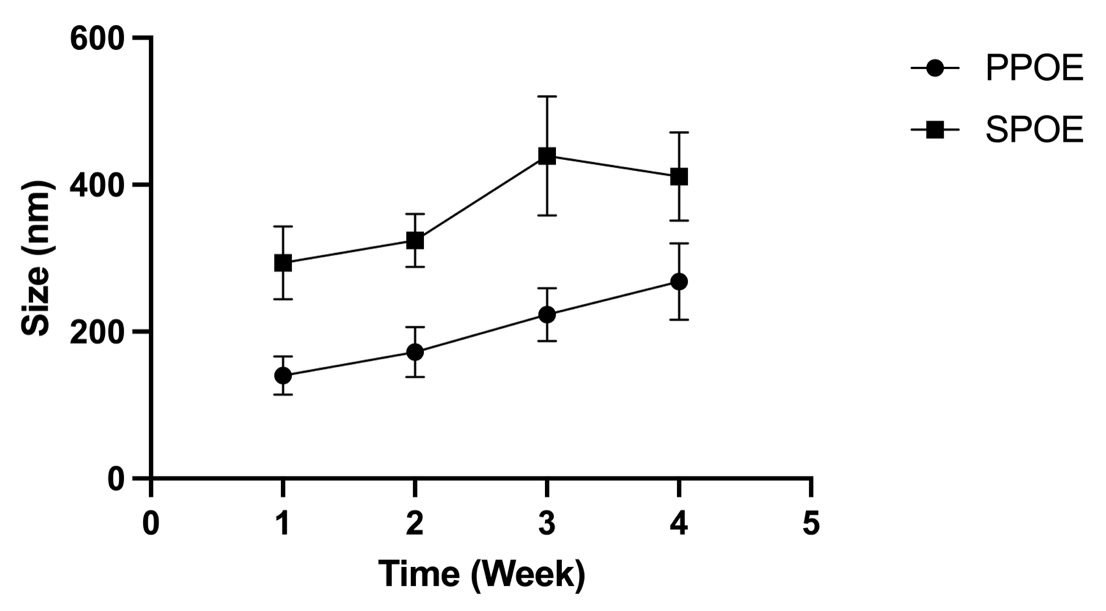


Figure S3. Short-term size depends on stability analyze. The samples were stored at room temperature.
